# PRKAR1B-AS2 Long Noncoding RNA Promotes Tumorigenesis, Survival, and Chemoresistance via the PI3K/AKT/mTOR Pathway

**DOI:** 10.3390/ijms22041882

**Published:** 2021-02-13

**Authors:** Abdelrahman M. Elsayed, Emine Bayraktar, Paola Amero, Salama A. Salama, Abdelaziz H. Abdelaziz, Raed S. Ismail, Xinna Zhang, Cristina Ivan, Anil K. Sood, Gabriel Lopez-Berestein, Cristian Rodriguez-Aguayo

**Affiliations:** 1 Department of Experimental Therapeutics, The University of Texas MD Anderson Cancer Center, Houston, TX 77054, USA; AMHamoda@mdanderson.org (A.M.E.); EBayraktar@mdanderson.org (E.B.); PAmero@mdanderson.org (P.A.); CIvan@mdanderson.org (C.I.); glopez@mdanderson.org (G.L.-B.); 2 Department of Pharmacology and Toxicology, Faculty of Pharmacy, Al-Azhar University, Cairo 11675, Egypt; salama@azhar.edu.eg (S.A.S.); abdelazizhamed@azhar.edu.eg (A.H.A.); raedismail@azhar.edu.eg (R.S.I.); 3 Department of Gynecologic Oncology and Reproductive Medicine, The University of Texas MD Anderson Cancer Center, Houston, TX 77030, USA; asood@mdanderson.org (A.K.S.); 4 Department of Medical and Molecular Genetics, Indiana University School of Medicine, Indianapolis, IN 46202, USA; xz48@iu.edu; 5 Center for RNA Interference and Non-Coding RNA, The University of Texas MD Anderson Cancer Center, Houston, TX 77054, USA

**Keywords:** lncRNA, PRKAR1B-AS2, AC147651.5, cisplatin resistance, PI3K/AKT/mTOR, ovarian cancer

## Abstract

Many long noncoding RNAs have been implicated in tumorigenesis and chemoresistance; however, the underlying mechanisms are not well understood. We investigated the role of PRKAR1B-AS2 long noncoding RNA in ovarian cancer (OC) and chemoresistance and identified potential downstream molecular circuitry underlying its action. Analysis of The Cancer Genome Atlas OC dataset, in vitro experiments, proteomic analysis, and a xenograft OC mouse model were implemented. Our findings indicated that overexpression of PRKAR1B-AS2 is negatively correlated with overall survival in OC patients. Furthermore, PRKAR1B-AS2 knockdown-attenuated proliferation, migration, and invasion of OC cells and ameliorated cisplatin and alpelisib resistance in vitro. In proteomic analysis, silencing PRKAR1B-AS2 markedly inhibited protein expression of PI3K-110α and abrogated the phosphorylation of PDK1, AKT, and mTOR, with no significant effect on PTEN. The RNA immunoprecipitation detected a physical interaction between PRKAR1B-AS2 and PI3K-110α. Moreover, PRKAR1B-AS2 knockdown by systemic administration of 1,2-dioleoyl-sn-glycero-3-phosphatidylcholine nanoparticles loaded with PRKAR1B-AS2–specific small interfering RNA enhanced cisplatin sensitivity in a xenograft OC mouse model. In conclusion, PRKAR1B-AS2 promotes tumor growth and confers chemoresistance by modulating the PI3K/AKT/mTOR pathway. Thus, targeting PRKAR1B-AS2 may represent a novel therapeutic approach for the treatment of OC patients.

## 1. Introduction

Ovarian cancer (OC) is the most fatal gynecologic tumor in women, accounting for approximately 14,000 deaths annually worldwide [1,2]. Currently, the standard treatment modality for OC relies on tumor debulking followed by a combination of platinum- and taxane-based chemotherapy [3,4,5,6]. Among these regimens, platinum is the most commonly used antineoplastic agent in OC. Despite initial promising response, most patients with OC experience relapse due to the emergence of platinum resistance [6]. Thus, there is an urgent need to explore additional targets implicated in the development of chemoresistance. The mechanisms underlying platinum resistance are multifactorial and generally include reduced DNA binding capacity, stimulation of DNA repair mechanisms, impaired expression of transporters, and altered expression of genes implicated in cell death pathways, such as BCL-2, p53, and PI3K/AKT/mTOR [7,8,9].

Phosphoinositide 3-kinases (PI3Ks) are members of the conserved lipid kinase family that exert their action by phosphorylating the 3′-hydroxyl group of phosphoinositides [10]. The PI3K family comprises four different classes, I, II, III, and IV, and only class I has been implicated in human cancers [10]. Class I is further subdivided into class IA and IB. Class IA is a heterodimer composed of a regulatory subunit (p85α, p55α, p50α, p85β, and p55γ) and a catalytic subunit (p110α, p110β, and p110δ) [11]. The PI3K/AKT/mTOR signaling cascade is a prototypic pathway that regulates diverse biological functions including proliferation, survival, apoptosis evasion, and protein synthesis [12,13]. Activation of this signaling pathway is provoked by binding of a growth factor ligand to its corresponding tyrosine kinase receptors, leading to recruitment of PI3K with subsequent phosphorylation of phosphatidylinositol-3,4-bisphosphate (PIP_2_) to produce phosphatidylinositol-3,4,5-triphosphate (PIP_3_). PIP_3_ acts as a plasma membrane docking site for phosphoinositide-dependent kinase 1 (PDK1) and protein kinase B (AKT), with subsequent phosphorylation of AKT at T308 residue [12]. Threonine phosphorylation of AKT induces sequential phosphorylation of downstream molecules, tuberous sclerosis complex 1 and 2 (TSC1/2), and mammalian target of rapamycin (mTOR), resulting in the activation of mTOR complex 1 (mTORC1). Following activation, mTORC1 induces phosphorylation of both ribosomal protein S6 kinases and 4E-binding protein 1, leading to increased cell proliferation and protein translation [12]. Hyperactivation of the PI3K/AKT/mTOR pathway is associated with increased tumor survival, drug resistance, and poor clinical outcome [12,13,14]. In OC, an analysis from The Cancer Genome Atlas (TCGA) showed that the PI3K/AKT/mTOR pathway is altered in approximately 34% of patients with high-grade serous carcinoma [15]. Activating mutations in *PIK3CA*, the PI3K 110α-encoding gene, have been identified in approximately 20% of patients with endometroid and clear-cell carcinoma, indicating that the PI3K/AKT/mTOR pathway plays a crucial role in OC [16].

It is well known that less than 2% of the human genome comprises protein-coding genes; most of the human genome is actively transcribed into noncoding RNAs [17]. Long noncoding RNAs (lncRNAs) are transcripts that surpass 200 nucleotides in length. Recent studies indicate that the human genome contains approximately 16,000 genes that encode for more than 28,000 lncRNA transcripts [18]. LncRNAs exert various biological functions such as epigenetic regulation of gene expression, RNA maturation and stability, protein translation, and post-translational modifications [19,20]. Mounting evidence indicates that dysregulation of lncRNAs is correlated with the development of multiple malignancies and chemoresistance [21,22,23,24,25,26]. In OC, several lncRNAs function as oncogenes and others might have tumor-suppressor effects, therefore modulating tumor growth and response to chemotherapy. Differential expression of lncRNAs has been detected between cisplatin-resistant and cisplatin-sensitive OC, providing evidence of the potential role of lncRNAs in the evolution of chemoresistance [27]. Although the effectiveness of some lncRNAs as potential therapeutic targets for relieving chemoresistance has been corroborated experimentally, efficient translation of these lncRNAs into clinical setting and approval of lncRNA-targeting strategies have not yet been achieved. The aim of the current study was to identify and characterize the role of the lncRNA PRKAR1B-AS2 in OC and to determine its potential as a therapeutic target in OC. Here, we report that PRKAR1B-AS2 lncRNA promotes carcinogenesis and chemoresistance, at least in part, by modulating the PI3K/AKT/mTOR axis. Thus, targeting PRKAR1B-AS2 may represent a potential therapeutic approach for OC.

## 2. Results

### 2.1. PRKAR1B-AS2 lncRNA Is Overexpressed in OC and Associated with Poor Survival

**Figure 1:**
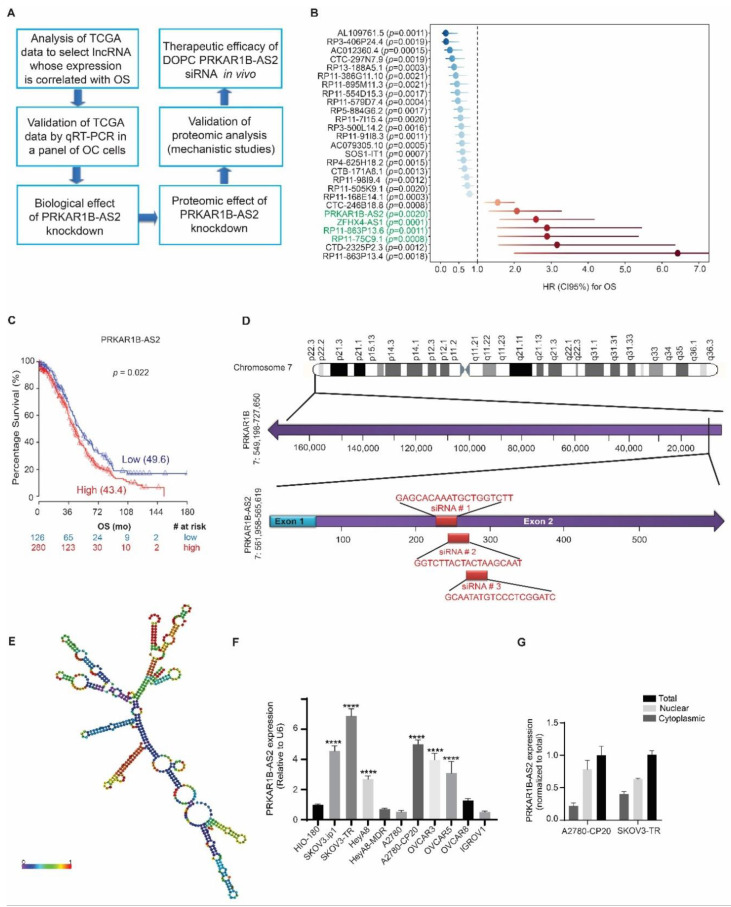
Protein Kinase CAMP-Dependent Type I Regulatory Subunit Beta (PRKAR1B-AS2) long noncoding RNA (lncRNA) is overexpressed in ovarian cancer (OC) and correlated with poor overall survival (OS). (**A**) Schematic diagram showing the workflow for the current study. (**B**) Forest plot showing the result of multivariable Cox regression analysis for the lncRNAs contributing to OS. The vertical line is the “line of null effect.” Each point represents the hazard ratio (HR). Each horizontal line represents the 95% confidence interval (CI). PRKAR1B-AS2 is an independent predictor for shorter OS: HR = 2.1, 95% CI = 1.3–3.28. (**C**) Kaplan–Meier plot generated with a cutoff of 0.31, log-rank *p* = 0.02. (**D**) Genomic location of PRKAR1B-AS2. PRKAT1B-AS2 resides at chromosome 7 in the antisense direction of its protein-coding gene, PRKAR1B. The lower panel shows the sequences and targets of the 3 independent siRNAs (highlighted in red). (**E**) Predicted 2-dimensional (2D) model for the secondary structure of PRKAR1B-AS2. The color code indicates base-pairing probabilities in a scale from 0 to 1 (red means the highest and violet means the lowest probability). (**F**) Quantitative reverse transcription PCR data showing PRKAR1B-AS2 expression in a panel of OC cell lines and the transformed noncancerous cell line HIO-180. Data are presented as means ± SD (**** *p* < 0.0001) (**G**) Subcellular localization of PRKAR1B-AS2 illustrating that PRKAR1B-AS2 is localized in both the cytoplasm and nucleus with a higher nuclear fraction. Data are normalized to total PRKAR1B-AS2.

The fact that various lncRNAs are implicated in tumorigenesis and chemoresistance given their differential overexpression in cancer has prompted us to investigate the role of lncRNAs in OC. We identified and studied the lncRNA of interest following the workflow illustrated in Figure 1A.

We analyzed data from TCGA to identify clinically significant lncRNAs whose expression is correlated with overall survival (OS) of OC patients. We identified 27 lncRNAs that were clinically associated with OS. Of those, 7 lncRNAs showed a negative correlation with OS (Figure 1B). Next, we ruled out 3 lncRNAs that have more than one RNA-binding domain according to the data published by Li. et al. [28]. We further validated the expression of the remaining 4 lncRNAs in OC by performing quantitative reverse transcription PCR and observed that only 2 lncRNAs, PRKAR1B-AS2, and RP11-863P13.6 were highly expressed in OC cells. We then selected PRKAR1B-AS2 to characterize in the current study, whereas the data generated for RP11-863P13.6 will be considered in another study. As mentioned above, our analysis of TCGA data revealed that PRKAR1B-AS2 overexpression is associated with shorter OS in OC (hazard ratio = 2.1, *p* = 0.02233; Figure 1C). PRKAR1B-AS2, also known as AC147651.5, is an antisense noncoding RNA that resides at chromosome 7: 561,958-565,619 and is transcribed in an opposite direction to its protein-coding gene, PRKAR1B (Figure 1D). The transcript is composed of 572 nucleotides, and it can fold at certain sequences to form a secondary structure (Figure 1E and Figure S1A). We next validated the expression level of PRKAR1B-AS2 in OC by screening a panel of 10 OC cell lines and one transformed noncancerous cell line, HIO-180. In this panel, we included high-grade OC cell lines and other histological subtypes as well. Our data revealed that PRKAR1B-AS2 expression is significantly higher in SKOV3.ip1, SKOV3-TR, HeyA8, A2780-CP20, OVCAR3, and OVCAR5 relative to HIO-180. Accordingly, SKOV3-TR and A2780-CP20-resistant cells were selected for further experiments because they showed the highest expression of PRKAR1B-AS2 (Figure 1F). The subcellular localization of lncRNAs is of biological importance because it can indicate function. Thus, we next determined the subcellular localization of PRKAR1B-AS2 using the PARIS kit. We observed that PRKAR1B-AS2 is localized in both cytoplasmic and nuclear compartments with a relatively higher level in the nuclear compartment (Figure 1G).

### 2.2. Silencing PRKAR1B-AS2 Inhibits Cell Viability and Improves the Response to Cisplatin In Vitro

**Figure 2:**
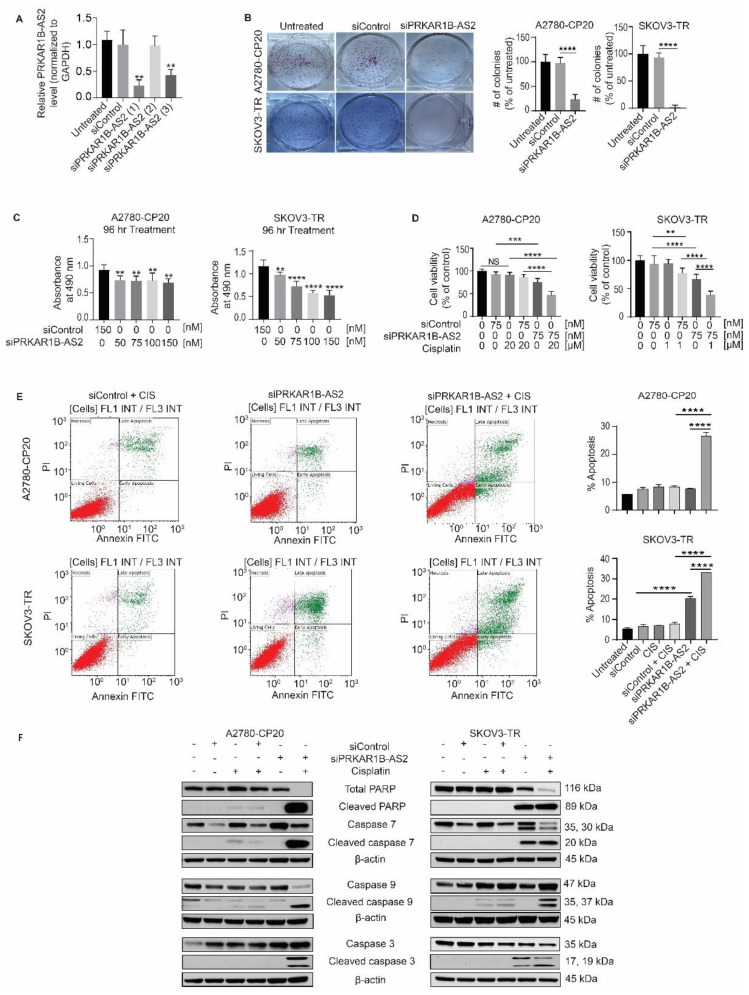
PRKAR1B-AS2 knockdown reduces cell viability and enhances cisplatin cytotoxicity in vitro. (**A**) PRKAR1B-AS2 expression level following transfection with 3 different siRNA sequences. (**B**) Colony formation assay results after transfecting A2780-CP20 and SKOV3-TR cells with either control siRNA or PRKAR1B-AS2 siRNA. Results were obtained by counting the number of colonies using Image J 1.53e software. (**C**) Cell viability results after transfecting A2780-CP20 and SKOV3-TR cells with 4 different concentrations of PRKAR1B-AS2 siRNA. (**D**) Cell viability results showing the effect of PRKAR1B-AS2 knockdown on cisplatin-induced cytotoxicity. (**E**) Annexin V/PI flow cytometry analysis illustrating that PRKAR1B-AS2 knockdown markedly exacerbates cisplatin (CIS)-induced apoptosis in A2780-CP20 and SKOV3-TR cells. (**F**) Western blot analysis of apoptosis-related parameters after treatment with PRKAR1B-AS2 siRNA, cisplatin, or PRKAR1B-AS2 siRNA combined with cisplatin in A2780-CP20 and SKOV3-TR cells. Combined treatment markedly increased the protein level of cleaved caspase 3, 7, 9, and cleaved PARP compared with cisplatin alone. β-actin was used as a loading control. Data are presented as means ± SD (** *p* < 0.01, *** *p* < 0.001, **** *p* < 0.0001). All experiments were independently performed at least 2 or 3 times.

To identify the biological roles of PRKAR1B-AS2 in OC, we established 3 independent PRKAR1B-AS2 targeting small interfering RNAs (siRNAs) and selected the one that produced the most potent silencing effect. Transfecting the cells with 3 independent siRNAs reduced PRKAR1B-AS2 expression by approximately 77% (*p* = 0.0011), 2% (*p* = 0.9998), and 57% (*p* = 0.009) compared with control siRNA. Control siRNA is a predesigned specific siRNA sequence that does not show any homology to the known human gene sequences (Figure 2A). Accordingly, PRKAR1B-AS2 siRNA-1 was selected for further experiments since it had the most potent silencing effect. Next, we aimed to evaluate the biological effect of silencing PRKAR1B-AS2 on 2 different OC cell lines, A2780-CP20 and SKOV3-TR, which showed the highest PRKAR1B-AS2 expression among the 10 cell lines examined. To determine the effect of PRKAR1B-AS2 knockdown on cell viability, we used the colony formation assay, which showed that PRKAR1B-AS2 knockdown significantly reduced the number of colonies (A2780-CP20: 23%, *p* < 0.0001; SKOV3-TR: 2%, *p* < 0.0001 compared with control siRNA; Figure 2B). To corroborate the ability of PRKAR1B-AS2 to regulate proliferation, we performed the MTS (3-(4,5-dimethylthiazol-2-yl)-5-(3-carboxymethoxyphenyl)-2-(4-sulfophenyl)-2H-tetrazolium) cell viability assay as an alternative supporting method. Concordantly, PRKAR1B-AS2 knockdown markedly decreased cell viability of both A2780-CP20 and SKOV3-TR cells (Figure 2C and Figure S1B,C). Next, we aimed to investigate the effect of PRKAR1B-AS2 silencing on cisplatin resistance. We first conducted an MTS aPlessay by treating A2780-CP20 and SKOV3-TR cells with escalating cisplatin concentrations (0.1, 0.5, 1, 5, 10, 20, 30, 40, and 50 µM) for different time intervals (24, 48, and 72 h) to select the appropriate concentration of cisplatin (Figure S2A,B). We then combined PRKAR1B-AS2 siRNA with cisplatin, and we observed that administration of PRKAR1B-AS2 siRNA significantly enhanced cisplatin sensitivity in both A2780-CP20 and SKOV3-TR cells, as manifested by reduced cell viability (Figure 2D).

We further explored whether PRKAR1B-AS2 knockdown induces apoptosis by conducting annexin V/PI flow cytometry analysis. Our findings indicated that PRKAR1B-AS2 knockdown markedly increased the percentage of apoptosis in SKOV3-TR cells (21%, *p* < 0.0001) but not in A2780-CP20 cells. Furthermore, combined treatment with PRKAR1B-AS2 siRNA and cisplatin significantly enhanced apoptosis in both A2780-CP20 cells (27%, *p* < 0.0001) and SKOV3-TR cells (33%, *p* < 0.0001) compared with the corresponding control groups (Figure 2E). Concordantly, the enhancement of cisplatin-induced apoptosis following PRKAR1B-AS2 knockdown was associated with increased expression of cleaved caspase 3, 7, and 9 and cleaved PARP (Figure 2F). In conclusion, these data revealed that PRKAR1B-AS knockdown reduces cell viability and ameliorates cisplatin resistance in both A2780-CP20 and SKOV3-TR OC cells.

### 2.3. PRKAR1B-AS2 Knockdown Suppresses Migration and Invasion of OC Cells In Vitro

**Figure 3:**
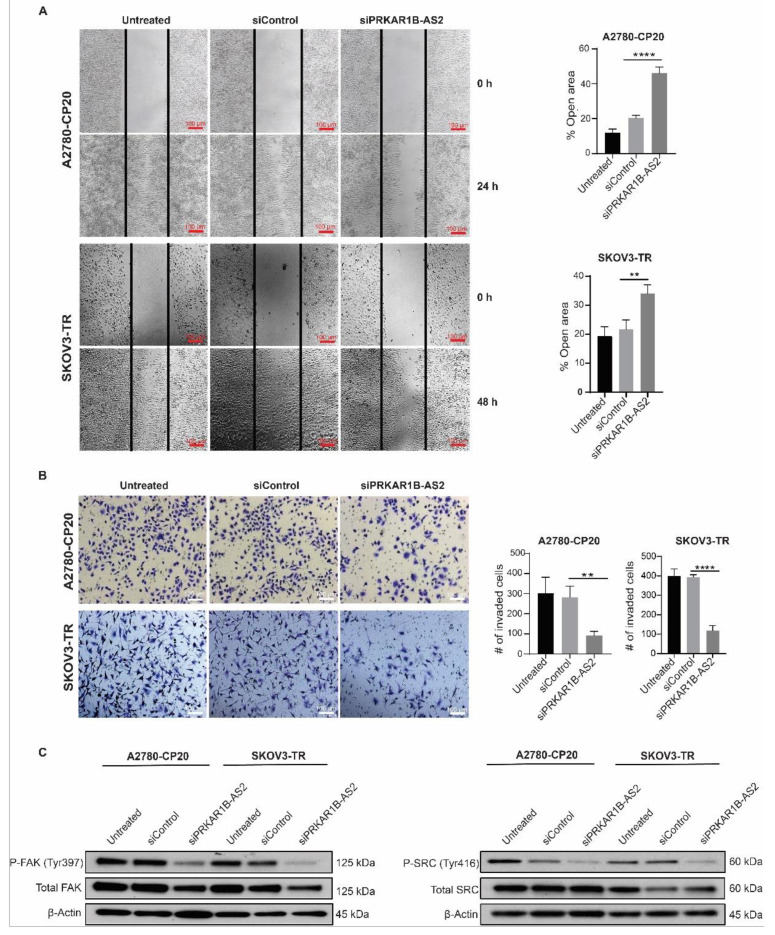
Silencing PRKAR1B-AS2 inhibits migration and invasion in vitro. (**A**) A2780-CP20 (8 × 10^4^) or SKOV3-TR (2 × 10^5^) cells were transfected with PRKAR1B-AS2 or control siRNA and subjected to the wound healing assay. Cells were detected under the microscope at 0 h and then at 6-h intervals until the control cells showed complete wound healing. The open areas were calculated using Image J 1.53e software and plotted using GraphPad Prism 8.0.0 software. (**B**) A2780-CP20 (8 × 10^4^) or SKOV3-TR (2 × 10^5^) cells were transfected with PRKAR1B-AS2 or control siRNA for 72 h and then seeded on serum-free Matrigel inserts for 24 h. Invading cells were analyzed by bright field microscopy and the number of invading cells was calculated using Image J software. (**C**) Protein expression levels of p-SRC (Tyr416), SRC, p-FAK (Tyr861), and FAK were validated by Western blot analysis after treating the cells with either PRKAR1B-AS2 or control siRNA. β-actin was used as a loading control. Data represent means ± SD of 3 independent experiments (** *p* < 0.01, **** *p* < 0.0001). All experiments were independently performed 3 times.

Because we observed that PRKAR1B-AS2 silencing reduces cell viability, we set out to determine whether PRKAR1B-AS2 knockdown affects the metastatic potential of OC cells. We performed the scratch wound healing assay in both A2780-CP20 and SKOV3-TR cells to investigate the effect of PRKAR1B-AS2 knockdown on migration. Results showed that silencing PRKAR1B-AS2 significantly increased the percentage of open area in A2780-CP20 cells (46%, *p* < 0.001) and SKOV3-TR cells (34%, *p* = 0.0054) compared with control siRNA (Figure 3A). We next used the Matrigel invasion assay to determine the consequence of PRKAR1B-AS2 knockdown on the invading capability of OC cells. As illustrated in Figure 3B, PRKAR1B-AS2 knockdown significantly decreased the number of invaded cells by approximately 67% (*p* = 0.007) in A2780-CP20 cells and 70% (*p* < 0.0001) in SKOV3-TR cells compared with control siRNA. Focal adhesion kinase (FAK) and steroid receptor coactivator (SRC) are nonreceptor tyrosine kinases that act downstream integrin transmembrane receptors. Phosphorylation of FAK and SRC at Tyr397 and Tyr416, respectively induces recruitment and activation of several signaling proteins ultimately leading to activation of migration, invasion, and metastasis [29]. We also performed Western blot analysis for p-SRC and p-FAK as an alternative approach to further validate our migration and invasion results. Our results revealed that silencing PRKAR1B-AS2 markedly inhibits the expression of p-SRC (Tyr416) and p-FAK (Tyr397) compared with control siRNA in both A2780-CP20 and SKOV3-TR cells (Figure 3C). Collectively, our findings indicated that silencing PRKAR1B-AS2 mitigates both the migratory and invading capability of OC cells.

### 2.4. PRKAR1B-AS2 Regulates the PI3K/AKT/mTOR Signaling Pathway

**Figure 4:**
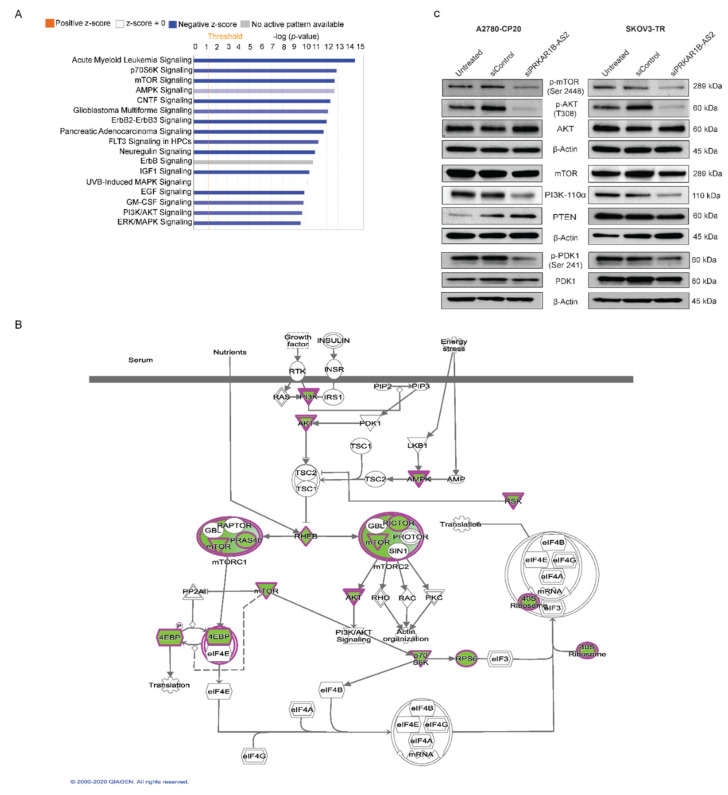
Proteomic characterization of PRKAR1B-AS2 in A2780-CP20 OC cells. (**A**) A graph generated by Qiagen Ingenuity Pathway Analysis software, using reverse phase protein array (RPPA) data, showing the most significantly altered signaling pathways following treating A2780-CP20 cells with either control or PRKAR1B-AS2 siRNA. The –log (*p*-value) 9.67 and 14.6 were used as cut-off points. (**B**) Ingenuity pathway analysis shows that PI3K/AKT/mTOR signaling pathway was markedly downregulated (*p* < 0.05) after PRKAR1B-AS2 knockdown. Green and encircled magenta colors refer to the downregulated proteins following PRKAR1B-AS2 silencing. (**C**) Validation of RPPA results by Western blotting. β actin was used as a loading control.

To have a better understanding of the role of PRKAR1B-AS2 in OC, we determined the potential downstream molecular targets whose protein expression was altered upon silencing PRKAR1B-AS2 using a high-throughput antibody-based functional proteomic analysis called reverse phase protein array (RPPA) in A2780-CP20. The RPPA platform is a robust, sensitive, and quantitative approach to validate key proteins by measuring the levels of proteins and their modifications such as phosphorylation and cleavage [30]. Ingenuity pathway analysis (IPA) identified multiple pathways that were significantly altered upon PRKAR1B-AS2 knockdown. Of those, the PI3K/AKT/mTOR canonical pathway was significantly (*p* < 0.05) downregulated after silencing PRKAR1B-AS2 (Figure 4A,B). Given the critical role of the PI3K/AKT/mTOR axis in tumorigenesis and chemoresistance, we hypothesized that PRKAR1B-AS2 induces its tumor-promoting action by regulating PI3K/AKT/mTOR circuitry. To test this hypothesis, we performed Western blot analysis to validate the protein expression levels following PRKAR1B-AS2 knockdown. Our results indicated that silencing PRKAR1B-AS2 markedly decreased the protein expression of PI3K-110α and abrogated the phosphorylation of AKT, PDK1, and mTOR (Figure 4C). Interestingly, the expression of phosphatase and tensin homolog (PTEN), a negative regulator for the PI3K/AKT/mTOR axis, was not significantly affected upon PRKAR1B-AS2 silencing (Figure 4C). To summarize, these results suggest that PRKAR1B-AS2 modulates the PI3K/AKT/mTOR axis by directly regulating PI3K-110α rather than having an indirect effect on PTEN.

**Figure 5:**
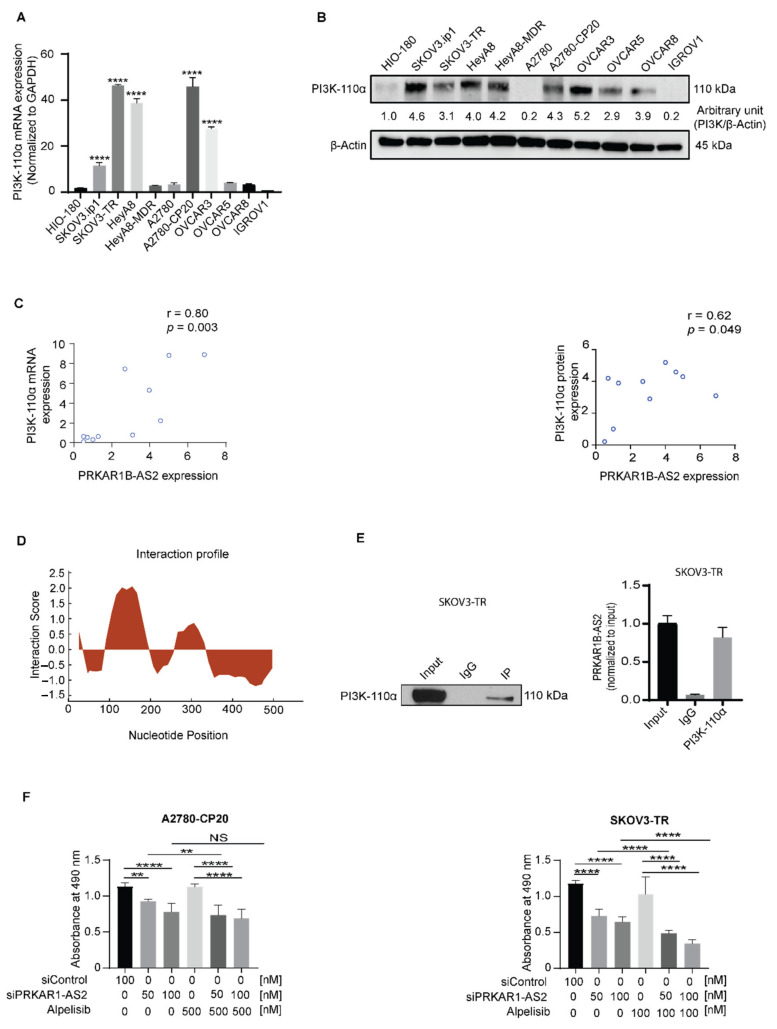
PRKAR1B-AS2 physically associates with PI3K-110α. (**A**) Quantitative reverse transcription PCR data showing the mRNA expression level of PI3k-110α in a panel of ovarian cancer (OC) cell lines and HIO-180 cells. (**B**) Detection of PI3K-110α protein expression in a panel of OC cell lines and HIO-180 cells. (**C**) Pearson correlation analysis of PI3K-110α mRNA and protein expression levels compared with PRKAR1B-AS2 expression in a panel of OC cell lines. (**D**) Computational analysis illustrating a high probability of PRKAR1B-AS2 and PI3K-110α protein interaction. (**E**) RNA immunoprecipitation assay results showing that the PRKAR1B-AS2 transcript is physically associated with PI3K-110α protein. (**F**) Effect of PRKAR1B-AS2 knockdown on alpelisib-induced cytotoxicity in both A2780-CP20 and SKOV3-TR OC cells. Data represent means ± SD of 3 independent experiments (** *p* < 0.01, **** *p* < 0.0001). NS = no statistical significance.

On the basis of the abovementioned findings, we postulated that PRKAR1B-AS2 expression is positively correlated with PI3K-110α expression in OC. To substantiate these findings, we first detected the basal mRNA and protein expression level of PI3K-110α in a panel of OC cell lines. Results showed that PI3K-110α is overexpressed in SKOV3.ip1, SKOV3-TR, HeyA8, HeyA8-MDR, A2780-CP20, and OVCAR3 cells compared with HIO-180 cells (Figure 5A,B). We next performed a Pearson correlation analysis of PI3K-110α mRNA and protein expression levels compared with PRKAR1B-AS2 expression and observed that PRKAR1B-AS2 expression is positively correlated with PI3K-110α at both the mRNA level (r = 0.80, *p* = 0.003) and protein level (r = 0.62, *p* = 0.049; Figure 5C).

According to our findings, we proposed that PRKAR1B-AS2 regulates PI3K/AKT/mTOR axis by physically binding to PI3K-110α. Thus, we performed an interaction profile bioinformatics analysis to determine whether PRKAR1B-AS2 interacts with PI3K-110α using RPISeq (http://pridb.gdcb.iastate.edu/RPISeq/; accessed on 23 July 2020) and catRAPID Fragments (http://s.tartaglialab.com/page/catrapid_group; accessed on 23 July 2020) software. Our findings showed a high propensity for PRKAR1B-AS2 lncRNA and PI3K-110α protein interaction (Figure 5D). We next attempted to validate the in silico findings via RNA immunoprecipitation (RIP) assay. First, we validated the efficiency of PI3K-110α immunoprecipitation by performing immunoblot analysis (Figure 5E, left panel). Next, we carried out quantitative reverse transcription PCR, and we observed that the PRKAR1B-AS2 RNA expression level was higher in PI3K-110α immunoprecipitation compared with IgG immunoprecipitation (Figure 5E, right panel). These data indicated that there is a physical interaction between PI3K-110α protein and PRKAR1B-AS2 lncRNA.

Our finding that PRKAR1B-AS2 regulates the PI3K-110α/AKT/mTOR signaling cascade prompted us to test the effect of PRKAR1B-AS2 knockdown on response to alpelisib, a selective inhibitor of PI3K-110α approved for the treatment of PIK3CA-mutated solid tumors, in both A2780-CP20 and SKOV3-TR cells. We first tested escalating concentrations of alpelisib at 3 different time intervals to select the appropriate concentration of alpelisib (Figure S2C,D). Our findings revealed that addition of 50 nM PRKAR1B-AS2 siRNA significantly enhanced alpelisib-induced cytotoxicity in A2780-CP20 and SKOV3-TR cells compared with their corresponding controls. However, the addition of 100 nM PRKAR1B-AS2 siRNA did not improve the response to alpelisib in A2780-CP20 cells (Figure 5F). These data indicated that silencing PRKAR1B-AS2 improves the response to alpelisib.

### 2.5. PRKAR1B-AS2 Silencing Improves OC Sensitivity to Cisplatin In Vivo

To confirm our in vitro findings, we investigated whether the siRNA-mediated silencing of PRKAR1B-AS2 produces effective antitumor activity in an orthotopic mouse model of cisplatin-resistant OC. In this experiment, we evaluated the effect of PRKAR1B-AS2-siRNA systemic delivery via 1,2-dioleoyl-sn-glycero-3-phosphocholine (DOPC) nanoliposomes to silence PRKAR1B-AS2 expression in vivo and to sensitize resistant tumors to cisplatin. A week following injecting mice with A2780-CP20 (cisplatin-resistant) cells (1 × 10^6^), mice were randomly divided into 4 treatment groups (n = 10 mice per group): (1) DOPC-control siRNA, (b) DOPC-PRKAR1B-AS2-siRNA, (c) DOPC-control siRNA + cisplatin, and (d) DOPC-PRKAR1B-AS2-siRNA + cisplatin. All siRNA treatments were administered intravenously twice weekly, and cisplatin was given intraperitoneally once weekly (Figure S3).

**Figure 6:**
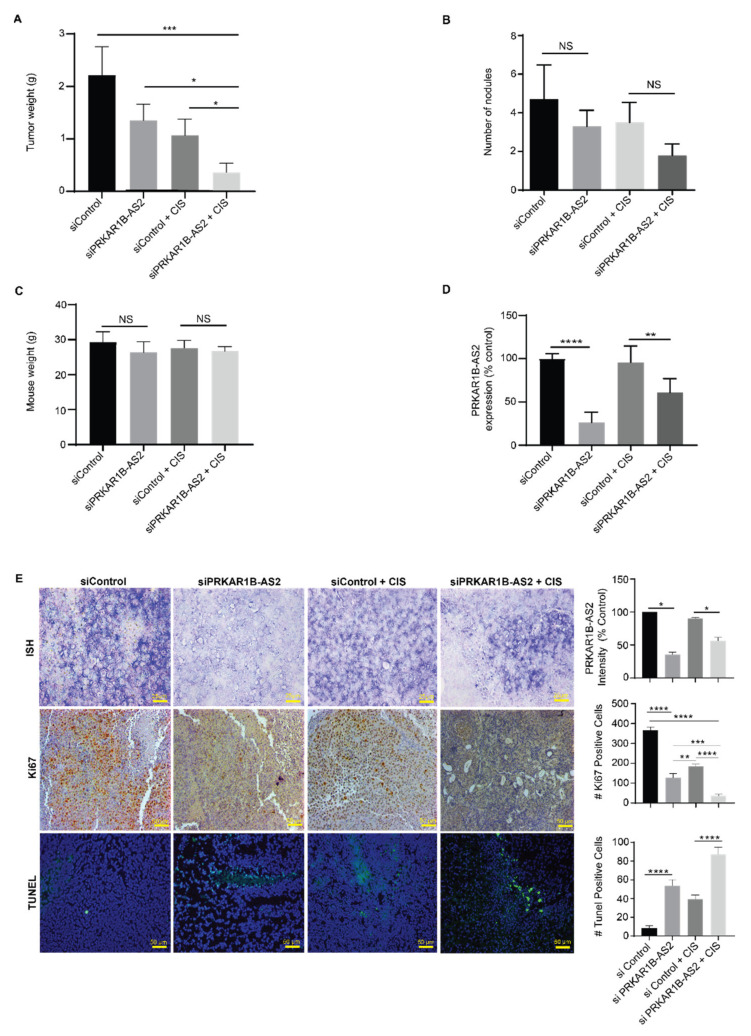
Systemic administration of PRKAR1B-AS2 liposomal nanoparticles reduces tumor growth and ameliorates cisplatin resistance in a xenograft mouse model. (**A**) Administration of DOPC-PRKAR1B-AS2-siRNA + cisplatin (CIS) significantly reduced tumor growth compared with DOPC-control siRNA or DOPC-control siRNA + cisplatin. (**B**) Effect of siRNA + cisplatin on the number of nodules. (**C**) Effect of siRNA + cisplatin on mouse weight. (**D**) Quantitative reverse transcription PCR analysis showing the relative expression of PRKAR1B-AS2 after subjecting mice to siRNA + cisplatin. (**E**). In situ hybridization (ISH), immunohistochemistry, and immunofluorescence analyses showing the effect of siRNA + cisplatin on PRKAR1B-AS2–positive, Ki67-positive, and terminal deoxynucleotidyl transferase dUTP nick-end labeling (TUNEL)-positive cells, respectively. Data are presented as means ± SD of 3 independent experiments (NS = no statistical significance, (*p* > 0.05, * *p* < 0.05, ** *p* < 0.01, *** *p* < 0.001, **** *p* < 0.0001).

Administration of DOPC-PRKAR1B-AS2-siRNA alone or DOPC-control siRNA + cisplatin reduced tumor weight by about 41% (*p* = 0.09) and 55% (*p* = 0.03), respectively, compared with DOPC-control siRNA alone. Administration of DOPC-PRKAR1B-AS2-siRNA + cisplatin significantly decreased tumor weight by 74% (*p* = 0.03) compared with PRKAR1B-AS2-siRNA alone and by 67% (*p* = 0.018) compared with DOPC-negative control siRNA + cisplatin (Figure 6A). Although the combined treatment reduced the number of nodules, this was not statistically significant compared with other treatment groups (Figure 6B). None of the treatments significantly affected mouse weight, indicating that the treatment has no marked toxic effect (Figure 6C).

To test the efficiency of liposomal nanoparticles in delivering siRNA to the tumor target site, we performed quantitative reverse transcription PCR to determine PRKAR1B-AS expression level following administration of DOPC-control siRNA or DOPC-PRKAR1B-AS2-siRNA. As shown in Figure 6D, silencing PRKAR1B-AS2 markedly inhibited the expression level of PRKAR1B-AS2 in mouse tissues compared with control. Interestingly, mice treated with PRKAR1B-AS2 siRNA + cisplatin showed a higher expression level of PRKAR1B-AS2 compared with mice treated with PRKAR1B-AS2 siRNA alone. The expression level of PRKAR1B-AS2 in mouse tissues following treatment with an siRNA + cisplatin was further confirmed by implementing in situ hybridization analysis (Figure 6E, upper panel).

Given the role of Ki67 as a proliferative biomarker, we performed Ki67 immunohistochemical analysis to determine whether PRKAR1B-AS2 knockdown affects proliferation in mouse tissues. Our findings revealed that mice treated with DOPC-PRKAR1B-AS2-siRNA and DOPC-PRKAR1B-AS2-siRNA + cisplatin showed a 65% decrease (*p* < 0.001) and 80% decrease (*p* < 0.001), respectively, in the number of Ki67-positive cells compared with their corresponding controls (Figure 6E, middle panel). To investigate the effect of PRKAR1B-AS2 silencing on apoptosis in vivo, we performed the terminal deoxynucleotidyl transferase dUTP nick-end labeling (TUNEL) assay. Our results revealed that mice treated with DOPC-PRKAR1B-AS2-siRNA or DOPC-PRKAR1B-AS2-siRNA + cisplatin had a significant increase in the number of apoptotic cells compared with their corresponding controls (Figure 6E, lower panel). In summary, these findings demonstrated that PRKAR1B-AS2 knockdown suppresses tumor growth and sensitizes the response to cisplatin in vivo.

## 3. Discussion

**Figure 7:**
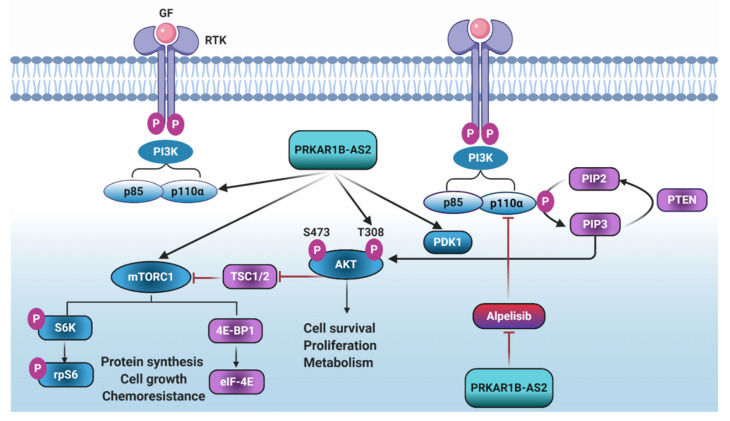
Schematic illustration showing activation of the phosphoinositide 3-kinase (PI3K)/protein kinase B (AKT)/mammalian target of rapamycin (mTOR) pathway and how PRKAR1B-AS2 regulates it. Specific growth factor (GF) binds to its cognate receptor tyrosine kinase (RTK) leading to autophosphorylation of the tyrosine kinase domain and recruitment of PI3K to the inner layer of cell membrane. The catalytic subunit of PI3K, in turn, induces phosphorylation of phosphatidylinositol bisphosphate (PIP2) to produce the second messenger phosphatidylinositol triphosphate (PIP3). PIP3 and phosphoinositide-dependent kinase 1 (PDK1) activate protein kinase B (AKT) via phosphorylation at threonine residue (T308), resulting in sequential phosphorylation and activation of the downstream targets mammalian target of rapamycin (mTOR), S6 kinase (S6K), S6 ribosomal protein (rpS6), and 4E-binding protein 1 (4E-BP1). Activation of this pathway stimulates protein synthesis and proliferation and confers chemoresistance. PRKAR1B-AS2 induces PI3K-110α expression and phosphorylation of PDK1, AKT, and mTOR and therefore activating proliferation and conferring cisplatin and alpelisib resistance.

The main findings from this study are that overexpression of PRKAR1B-AS2 is negatively correlated with overall survival in OC patients. Furthermore, PRKAR1B-AS2 promotes tumorigenesis and chemoresistance, at least in part, by positively regulating PI3K/AKT/mTOR axis (Figure 7).

One of the major advantages of our study is that we used an unbiased approach to explore the potential targets for PRKAR1B-AS2. Through this approach, we were able to determine the pathways altered upon silencing PRKAR1B-AS2 in the A2780-CP20 OC cell line. Accordingly, mTOR and PI3K/AKT signaling pathways were significantly downregulated after PRKAR1B-AS2 knockdown. Aberrant activation of the PI3K/AKT/mTOR pathway, a major pathway that regulates proliferation, apoptosis, and cell cycle progression, is implicated in the development of several cancers [31,32]. The clinically relevant data obtained from TCGA have shown that hyperactivation of the PI3K/AKT/mTOR pathway occurs in approximately 60% of OC patients in whom multiple activating PIK3CA and PIK3CB mutations have been identified [32,33,34,35]. Here, we show that silencing PRKAR1B-AS2 markedly inhibited PI3K-110α protein expression and abrogated the phosphorylation of PDK1, AKT, and mTOR with no significant effect on PTEN in both A2780-CP20 and SKOV3-TR cells. SKOV3 cells harbor an amplifying mutation for PIK3CA, whereas A2780 is characterized by loss of PTEN function [36]. These data could explain why A2780-CP20 and SKOV3-TR cells show high expression of PRKAR1B-AS2 and PI3K-110α in our results. It was previously reported that HULC (highly upregulated in liver cancer) lncRNA promotes proliferation, invasion, and angiogenesis by regulating the PI3K/AKT/mTOR axis in glioma [37]. Likewise, antisense noncoding RNA in the INK4 locus (ANRIL) enhances proliferation and tumorigenesis by epigenetically silencing miR-99a/miR-449a, and consequently upregulating mTOR and cyclin-dependent kinase 6 downstream pathways in gastric cancer [38]. Considering these findings, we suggest that the inhibition of the PI3K/AKT/mTOR signaling pathway could attribute to the antitumor effect of PRKAR1B-AS2 knockdown. PTEN acts as a negative regulator for this pathway by dephosphorylating PIP_3_. Partial loss of PTEN function can promote and accelerate tumorigenesis in multiple cancers [39]. The finding that PRKAR1B-AS2 knockdown did not enhance protein expression of PTEN may explain how PRKAR1B-AS2 regulates PI3K/AKT/mTOR molecular circuitry via direct regulation of PI3K-110α rather than indirect action mediated by PTEN. Dysregulation of the PI3K/AKT axis enhances cancer cell invasiveness and facilitates tumor progression [40]. Combined inhibition of PI3K and SRC was reported to exhibit synergistic suppression of invasion and proliferation in renal cell carcinoma [41]. Concordantly, we suggest that PRKAR1B-AS2 knockdown inhibits migration and invasion in OC cells, at least in part, by blocking the expression of PI3K-110α and p-SRC.

LncRNA expression levels can be inhibited through RNA interference technology; however, the delivery of nucleic-acid-based therapy into specific target sites is a challenge owing to the secondary structure or intracellular localization of lncRNAs. One of the most successful platforms for siRNA delivery is a neutral liposomal formulation based on DOPC that delivers siRNA with a greater efficacy compared with cationic liposomes [42]. Indeed, our findings revealed that PRKAR1B-AS2 silencing by DOPC-siRNA-attenuated tumor growth and improved the response to cisplatin in vivo. Furthermore, systemic administration of cisplatin induced the expression level of PRKAR1B-AS2 in mouse tissues. It was previously reported that cisplatin-resistant OC cell lines, including A2780-CP20, induce the expression of oncogenes and DNA repair enzymes in response to cisplatin treatment [43]. Furthermore, cisplatin treatment induced the expression of PANDAR lncRNA with a subsequent augmentation of tumor growth and cisplatin resistance in OC [44]. Similarly, we suggest that A2780-CP20-resistant mouse model responded to cisplatin treatment by inducing the expression of PRKAR1B-AS2. The emergence of platinum and/or taxane resistance is the major cause of relapse and mortality in OC [45]. Multiple preclinical studies indicated that the emergence of chemoresistance in OC can be traced to hyperactivation of the PI3K/AKT/mTOR axis [46,47,48,49]. Consistent with these reports, our findings indicate that PRKAR1B-AS2 indirectly confers cisplatin resistance by activating the PI3K/AKT/mTOR signaling pathway. Alpelisib, a selective inhibitor of PI3K-110α, is approved for the management of PIK3CA-mutated, HER2-negative breast cancer [50,51]. The combination of alpelisib and olaparib has been shown to exhibit a synergistic effect against BRCA wild-type, platinum-resistant OC [52]. Although several PI3K inhibitors have been translated into the clinic, the emergence of resistance limits their clinical use. Thus, PI3K inhibitors are combined with other chemotherapy to improve the overall clinical outcome [53]. Here, we report that silencing PRKAR1B-AS2 enhances alpelisib-induced cytotoxic effect on OC cells. These data could pave the path toward harnessing PRKAR1B-AS2 as a potential target for improving the response to PI3K inhibitors in OC.

Recently, it was reported that PI3K-110α has 2 protein pools, A and B. Pool A resides mainly at the cytoplasm, whereas pool-B translocates into the nucleus of only cancerous cells [54]. Mechanistically, using the RIP assay, we observed a physical association between PI3K-110α protein and PRKAR1B-AS2. Precisely how PRKAR1B-AS2 regulates PI3K-110α warrants further investigation. However, we can rule out transcriptional or post-transcriptional regulation since PRKAR1B-AS2 knockdown did not significantly alter the PIK3CA mRNA expression level. These data, together with the finding that PRKAR1B-AS2 is localized in both the cytoplasm and the nucleus, with a higher nuclear fraction, might indicate that PRKAR1B-AS2 could directly regulate PI3K-110α at either a translational or post-translational level. Nuclear lncRNAs plausibly regulate gene expression by modulating chromatin remodeling, transcription, and post-transcriptional modifications [55,56,57,58]. In contrast, cytoplasmic lncRNAs regulate mRNA turnover, sponging of certain cytosolic factors, protein translation, and stability [59]. On the basis of these data, we conjecture that PRKAR1B-AS2 binds to both nuclear and cytoplasmic protein pools of PI3K-110α enhancing their stability and protecting them from degradation. This explanation is consistent with previous data showing that the lncRNAs LINRIS and GLCC1 promote tumor growth by inhibiting the degradation and enhancing the stability of oncogenic proteins IGF2BP2 and c-MYC, respectively [60,61].

In conclusion, PRKAR1B-AS2 promotes tumor growth and survival in OC via regulating PI3K/AKT/mTOR pathway. Furthermore, PRKAR1B-AS2 physically associates with and directly regulates PI3K-110α protein. Thus, targeting PRKAR1B-AS2 might represent a potential approach for relieving cisplatin and alpelisib resistance in OC.

## 4. Materials and Methods

### 4.1. OS Analysis of lncRNA Expression in TCGA OC Cohort

The OS analysis for TCGA data was performed in R (version 3.4.1) (http://www.r-project.org/; accessed on 20 June 2017) and the level of statistical significance for any hypothesis test applied was set to 0.05. We downloaded clinical information for TCGA patients with ovarian serous adenocarcinoma from cBioPortal (http://www.cbioportal.org/; accessed on 20 June 2017). From The Atlas of Non-coding RNA in Cancer (https://bioinformatics.mdanderson.org/public-software/tanric/; accessed on 20 June 2017), we downloaded lncRNA RPKM expression quantification data for primary tumors. We ended up with a cohort of 406 patients with lncRNA expression and clinical information available. We analyzed the association of gene expression and other clinical parameters available (age and tumor stage) with OS using a Cox proportional hazards model. In the multivariable regression model, only factors that were statistically significant in the univariate analyses were included (age and gene expression). We then compiled a forest plot summarizing the hazard ratios, 95% confidence intervals, and *p* values (calculated using the Wald test) for lncRNAs significantly associated with OS in the multivariable analysis. To visualize the survival difference for our gene of interest, PRKAR1B-AS2, we used the log-rank test to find the cutoff point for the most significant split (i.e., lowest *p* value) between the groups with the highest and lowest lncRNA levels. A Kaplan–Meier plot was generated using this cutoff. The number of patients at risk in the low- and high-lncRNA groups at different time points was presented at the bottom of the graph (Figure 1C). In brackets, we presented the median survival (in months) for each group.

### 4.2. LncRNA–Protein Interactions

We downloaded data for lncRNA protein-binding domains from a combinatorial analysis among the RBP-lncRNA interactome published by Li et al., in which 12,255 human lncRNAs with at least one binding site for RNA-binding proteins were analyzed [28]. We used 2 independent software programs (http://pridb.gdcb.iastate.edu/RPISeq/ and http://s.tartaglialab.com/; accessed on 23 July 2020) to determine the probability of PRKAR1B-AS2 lncRNA and PI3K-110α interaction.

### 4.3. Cell Culture, Reagents, and siRNA Transfection

Human OC cell lines HeyA8, SKOV3-ip1, A2780, HeyA8-MDR, SKOV3-TR, A2780-CP20, OVCAR3, OVCAR-5, OVCAR-8, and IGROV1, as well as immortalized normal ovarian epithelial cell line HIO180 were obtained from The University of Texas MD Anderson Cancer Center Characterized Cell Line Core Facility or ATCC (American Type Culture Collection, Manassas, VA, USA). The abovementioned cells were cultured in RPMI1640 medium (Gibco BRL, Rockville, MD, USA), supplemented with 10% fetal bovine serum and 1% penicillin/streptomycin solution. All cell lines were maintained in 5% CO_2_ and 95% air at 37 °C. All cell lines were screened for mycoplasma using the MycoAlert mycoplasma detection kit (Lonza). All experiments were conducted with cell cultures at 60–80% confluence. All siRNA transfections were performed with HiPerFect (Qiagen, USA) reagent using the forward transfection protocol from the manufacturer. Three independent sequences of PRKARB-AS siRNA and a negative control siRNA were used (Table 1).

### 4.4. RNA Extraction, Reverse Transcription, and Quantitative Real-Time PCR Analysis

Cells were collected using TRIzol (Invitrogen, Carlsbad, CA, USA) and RNA was isolated and purified based on the manufacturer instructions. Nuclear and cytoplasmic RNA was purified and isolated from OC cells using the PARIS kit (Thermo Fisher, Waltham, MA, USA). For detection of PRKAR1B-AS2 expression level, 1 μg of each total RNA was reverse-transcribed using the Superscript III One-Step Reverse Transcription-PCR System (Invitrogen, Carlsbad, CA, USA) according to the manufacturer’s protocol, under the following conditions: 37 °C for 60 min, 70 °C for 5 min, 42 °C for 20 min, and 85 °C for 5 min. The CFX 384 Real-Time PCR System (Bio-Rad, Hercules, CA, USA) was used to run all PCR reactions using iTaq Universal SYBR Green Super mix (Bio-Rad, Hercules, CA, USA). All primers were purchased from Sigma Aldrich (St. Louis, MO, USA) (Table 2). Thirty-nine cycles of amplification were conducted under the following conditions: melting at 95 °C for 3 min, annealing at 60 °C for 30 s, and melting curve at 65–95 °C in increments of 0.5 °C. PRKAR1B-AS2 data were normalized to the internal control small nuclear RNA RNU6B, PIK3CA mRNA was normalized to GAPDH, and RNA expression levels were determined using the 2^−ΔΔCT^ method.

### 4.5. Cell Viability Assay

Cell viability was assayed using the MTS reagent. Briefly, A2780-CP20 (1 × 10^3^/well) and SKOV3-TR (1.5 × 10^3^/well) cells were seeded in 96-well culture plates. Cells were treated with escalating concentrations of PRKAR1B-AS2 siRNA, cisplatin, or alpelisib at different time intervals to select the appropriate concentrations. Next, combined treatment with either PRKAR1B-AS2 siRNA + cisplatin or PRKAR1B-AS2 siRNA + alpelisib was administered by incubating the siRNA for 96 h and then adding the chemotherapy 48 h after initiating treatment with siRNA. At the end of the treatment schedule, 20 µL of MTS reagent was added to each well and the absorbance was recorded at 490 nm by a spectrophotometer (Bio-Rad Microplate Reader, Hercules, CA, USA).

### 4.6. Colony Formation Assay

Cells were plated onto 6-well plates (1×10^3^ cells/well for A2780-CP20 and 2 × 10^3^ cells/well for SKOV3-TR), transfected with control or PRKAR1B-AS2 siRNA, and incubated at 37 °C for 1–3 weeks to allow for colonies formation. At the end of the incubation, the colonies were washed once with phosphate-buffered saline, subjected to crystal violet (0.5% *w*/*v*) staining, and photographed. Image J 1.53e software was implemented to calculate the number of colonies per well. A colony was defined as consisting of at least 50 cells. Each experiment was performed in triplicate and repeated 3 times.

### 4.7. Wound Healing Assay

Cell migration was assessed by the wound healing assay. A2780-CP20 cells (1 × 10^5^ cells/well) and SKOV3-TR cells (2 × 10^5^ cells/well) were plated onto 6-well plates for 24 h before initiating control or PRKAR1B-AS2 siRNA transfection. Cells were incubated at 37 °C until reaching 100% confluence to form a monolayer. Next, an accurate scratch was performed in each cell monolayer using a 200-μL pipet tip. Cellular debris was taken out by washing with Hanks’ balanced salt solution (ThermoFisher Scientific, Carlsbad, CA, USA). Images were captured at 0, 12, 24, and 36 h (depending on cell line) after scratching using a phase-contrast Nikon eclipse TEμ0-U microscope (Carlsbad, CA, USA). The rate of migration inhibition was assessed by calculating the open area at the end of experiment relative to the open area at time zero. The obtained values were expressed as a percentage of the open area. Each experiment was performed in triplicate and repeated 3 times.

### 4.8. Invasion Assay

Cell invasiveness was investigated by Matrigen invasion assay. Transwell chambers were coated with Matrigel (BD Biosciences, San Jose, CA, USA) rich in extracellular matrix proteins. A2780-CP20 or SKOV3-TR cells transfected with control or PRKAR1B-AS2 siRNA were mixed with serum-free medium and added into the upper Matrigel-coated chambers (5.0 × 10^5^ cells/chamber). Fetal bovine serum-containing medium was added to the lower chambers to serve as a chemoattractant. The cells were incubated at 37 °C for 24 h. Following the incubation, the cells in the upper chamber were taken off using specific cotton swabs. Cells that invaded the lower chambers were stained using the Hema 3 staining set (Fisher Scientific, Carlsbad, CA, USA). Image J 1.53e software was then used to calculate the number of invaded cells. Each experiment was performed in triplicate and repeated 3 times.

### 4.9. Annexin V/PI Assay

The resistant A2780-CP20 and SKOV3-TR cell lines were transfected with control or PRKAR1B-AS2 siRNA for 96 h, followed by initiation of treatment with cisplatin after the first 48 h. At the end of treatment, cells were processed and stained with the FITC-Apoptosis and Propidium Iodide Detection Kit (BD Pharmingen, San Diego, CA, USA). Apoptosis was assessed by Gallios Cell Analyzer (Indianapolis, IN, USA) and Kaluza for Gallios 1.0.14029.14028 software according to the manufacturer’s recommended protocol.

### 4.10. Cell Extracts and Western Blot Analysis

Total cell extracts were prepared in standard RIPA buffer containing protease and phosphatase inhibitor cocktail (Roche, Basel, Switzerland). Lysates were centrifuged at 14,000× *g* for 20 min at 4 °C, and supernatants were collected. The protein concentration was determined using the Pierce (bicinchoninic acid) BCA protein assay kit according to the manufacturer protocol (Thermo Scientific, IL, USA). Following protein quantitation, samples were boiled at 100 °C for 5 min, loaded into SDS polyacrylamide gel, and transferred overnight to polyvinylidene difluoride membranes (Millipore Co, Cork, Ireland). The expression levels of selected proteins were determined using their corresponding primary antibodies followed by incubation with horseradish peroxidase–conjugated secondary antibodies (Table 3). Immunoblots were developed using HyGLO Chemiluminescent HRP Antibody Detection Reagent (Denville Scientific Inc, Metuchen, NJ, USA), and signals were recorded using either X-ray film or ChemiDoc Imaging System (Bio-Rad, CA, USA). β-actin was used as a loading control and the band intensities were quantified through densiometric analysis relative to β-actin.

### 4.11. RIP Assay

Magnetic beads were purified and incubated with either PI3K-110α antibody or normal rabbit IgG (control) for 30 min at room temperature to form a bead-antibody complex. A2780-CP20 and SKOV3-TR cells were subjected to lysis with RIP lysis buffer and incubated overnight with bead-antibody complexes at 4 °C to coimmunoprecipitate the desired proteins bound to RNAs. After incubation, Western blot analysis was performed to test the immunoprecipitation efficacy, and then coimmunoprecipitated RNAs were purified, extracted using phenol:chloroform:isoamyl alcohol, and subjected to quantitative reverse transcription PCR according to Magna RIP protocol (Millipore Sigma, Burlington, MA, USA).

### 4.12. RPPA Analysis

After A2780-CP20 cells were treated with control siRNA or PRKAR1B-AS2 siRNA, cells were washed with phosphate-buffered saline. Lysis buffer with protease/phosphatase inhibitors was added to prevent protein degradation and stabilize phosphorylation. Cell lysates were centrifuged at 14,000 rpm for 20 min at 4 °C. Pierce BCA protein assay kit (Thermo Scientific, IL, USA) was used to determine the protein content of samples according to the manufacturer protocol. Protein samples were adjusted to 1.0 μg/μL, denatured at 100 °C for 5 min, and stored at −80 °C until RPPA processing.

### 4.13. Liposomal Nanoparticle Preparation

PRKAR1B-AS2 siRNA was incorporated into DOPC liposomes for systemic administration as previously described [62,63]. DOPC and PRKAR1B-AS2 siRNA were mixed in the presence of excess tertiary butanol at a ratio of 1:10 *w*/*w*. Tween-20 was added to the mixture in a ratio of 1:19. The mixture was then subjected to vortex and lyophilization. Immediately before in vivo administration, this preparation was reconstituted in phosphate-buffered saline to have a final concentration of 200 μg/kg siRNA per injection.

### 4.14. OC Xenograft Mouse Model Studies

Female athymic nude mice were obtained from Taconic Farms. Animal studies were conducted according to the guidelines set forth by the American Association for Accreditation of Laboratory Animal Care and the US Public Health Service policy on Humane Care and Use of Laboratory Animals. All mouse studies were approved by The University of Texas MD Anderson Cancer Center Institutional Animal Care and Use Committee (00001010-RN02). The age of all mice was in the range of 6–8 weeks at the time of injection. A2780-CP20 cells were collected using trypsin-EDTA, neutralized with fetal bovine serum–containing medium, resuspended in Hanks’ balanced salt solution, and injected into nude mice (1 × 10^6^ cells/mouse). Nude mice bearing A2780-CP20 tumors were randomly divided into 4 groups (10 mice/group) and treated with the following: DOPC-negative control siRNA (200 μg/kg), DOPC-PRKAR1B-AS2-siRNA (200 μg/kg), DOPC-negative control siRNA (200 μg/kg) + cisplatin (4 mg/kg), or DOPC-PRKAR1B-AS2-siRNA (200 μg/kg) + cisplatin (4 mg/kg). Cisplatin was injected intraperitoneally once weekly, and siRNA was administered intravenously twice weekly. The dose of DOPC/siRNA was selected according to the data published earlier [62,63,64]. After 6 weeks of initiating treatment or once the mice became moribund, they were killed and tumors were harvested. Tumor weight and number and location of nodules were recorded. Tumor tissues were fixed in formalin for paraffin embedding, frozen in optimal cutting temperature medium for frozen slide preparation, or snap-frozen for lysate preparation.

### 4.15. Immunohistochemistry

Immunohistochemistry analysis for Ki67 was performed on 4-μm formalin-fixed paraffin-embedded epithelial cancer sections. After deparaffinization, slides were subjected to antigen retrieval using 1× Diva Decloaker (BioCare Medical, Pacheco, CA, USA) under a steamer. Endogenous peroxidases were blocked using 3% hydrogen peroxide in methanol. Nonspecific binding was blocked using a mixture of 5% normal horse serum and 1% normal goat serum in phosphate-buffered saline. Samples were then subjected to overnight incubation with primary antibody against Ki67 (1:200, NeoMarkers. Next, Goat anti-rabbit horseradish peroxidase (Jackson Immuno Research Laboratories, West Grove, PA, USA) diluted in blocking solution was added as a secondary antibody. The slides were analyzed individually using a bright field Nikon eclipse TEμ0-U microscope (CA, USA). The number of Ki67-positive cells was counted for each slide and plotted in GraphPad Prism 8.0.0 software to determine the statistical significance. The mean for each group was then plotted in the graph, while only one representative photo was presented.

### 4.16. TUNEL Assay

The TUNEL assay (Promega, Madison, WI, USA) was conducted on 4-μm sections of fresh frozen specimens embedded in optimal cutting temperature medium according to the manufacturer’s protocol. Fluorescence microscopy was used to determine the fragmented DNA of apoptotic cells. The number of apoptotic cells was then calculated by Image J 1.53e software.

### 4.17. In Situ Hybridization

Frozen tissue sections were first digested with 5 μg/mL proteinase K for 5 min at room temperature and were then loaded onto Ventana Discovery Ultra system (Ventana Medical Systems, Inc, Tucson, AZ, USA) for in situ hybridization analysis. The tissue slides were incubated with double-DIG labeled custom LNA probe for PRKAR1B-AS (Exiqon, Qiagen, Hilden, Germany) for 2 h at 55 °C. The digoxigenins were detected with a polyclonal anti-DIG antibody and alkaline phosphatase–conjugated second antibody (Ventana, Oro Valley, AZ, USA) using NBT-BCIP as the substrate. The double-DIG labeled control U6 snRNA probe was also from Exiqon.

### 4.18. Collection of Mouse Tumor Specimens for Quantitative Reverse Transcription PCR

Individual tumor pieces, each with an approximate size of 1 mm^3^, were dissected from three different mice per each group. Following homogenization, RNA was isolated and 1 mcg of total RNA was implemented to prepare the first strand cDNA. The expression of PRKAR1B-AS2 was then determined using CFX 384 Real Time PCR System (Bio-Rad, Hercules, CA, USA). Three experimental replicates for each sample were used to assemble the figure in GraphPad Prism 8.0.0 software to determine the statistical significance.

### 4.19. Statistical Analysis

The Student *t*-test (unpaired, 2-tailed) was used to compare independent samples from 2 different groups, and analysis of variance was utilized to compare samples from more than 2 groups. All statistical tests were 2-tailed and performed by GraphPad Prism 8.0.0 software. All data were presented as mean ± SD, and *p* < 0.05 was considered statistically significant.

## Figures and Tables

**Table 1:** Sequences of Protein Kinase CAMP-Dependent Type I Regulatory Subunit Beta (PRKAR1B-AS2) small interfering RNA (siRNA) used in the current study.

siRNA Name	Sequence (Sense) 5′ to 3′	Sequence (Antisense) 5′ to 3′
PRKAR1B-AS2 siRNA 1	GAGCACAAAUGCUGGUCUUdTdT	AAGACCAGCAUUUGUGCUCdTdT
PRKAR1B-AS2 siRNA 2	GGUCUUACUACUAAGCAAUdTdT	AUUGCUUAGUAGUAAGACCdTdT
PRKAR1B-AS2 siRNA 3	GCAAUAUGUCCCUCGGAUCdTdT	GAUCCGAGGGACAUAUUGCdTdT

**Table 2:** Sequences of Primers.

Gene Symbol	Forward Primer	Reverse Primer
PRKAR1B-AS2	5′-TTGGACACTGCCCATCTT-CC3′	5′-TGCAGCCACGGGATGTTT-AT3′
PRKAR1B-AS2	5′-CCTCGGATCCAAAGGAGA-GC-3′	5′-AAGATGGGCAGTGTCCAA-GG-3′
PIK3CA	5′-GAGTAACAGACTAGCTAGAGAC-3′	5′-AGAAAATCTTTCTCCTGCTC-3′
GAPDH	5′-GACAGTCAGCCGCATCTT-CT-3′	5′-GCGCCCAATACGACCAAA-TC-3′
U6 snRNA	5′-CTCGCTTCGGCAGCACA-3′	5′-GGAACGCTTCACGAATTT-GC-3′

**Table 3:** List of antibodies used.

Primary Antibody	Purchasing Company (Catalog #)	Dilution Factor	Secondary Antibody	Purchasing Company	Dilution Factor
PI3 Kinase p110α (C73F8) Rabbit mAb	Cell Signaling Technology, Danvers, MA, USA (4249)	1:1000	Anti-rabbit IgG, HRP-linked Antibody	Cell Signaling Technology, Danvers, MA, USA (7074)	1:2000
PI3 Kinase p110α Antibody	Cell Signaling Technology, Danvers, MA, USA (4255)	1:1000	Anti-rabbit IgG, HRP-linked Antibody	Cell Signaling Technology, Danvers, MA, USA (7074)	1:2000
Phospho-mTOR (Ser2448) Antibody	Cell Signaling Technology, Danvers, MA, USA (2971)	1:500	Anti-rabbit IgG, HRP-linked Antibody	Cell Signaling Technology, Danvers, MA, USA (7074)	1:2000
mTOR (7C10) Rabbit mAb	Cell Signaling Technology, Danvers, MA, USA (2983)	1:1000	Anti-rabbit IgG, HRP-linked Antibody	Cell Signaling Technology, Danvers, MA, USA (7074)	1:2000
Phospho-Akt (Ser473) (D9E) Rabbit mAb	Cell Signaling Technology, Danvers, MA, USA (4060)	1:400	Anti-rabbit IgG, HRP-linked Antibody	Cell Signaling Technology, Danvers, MA, USA (7074)	1:2000
Akt (pan) (40D4) Mouse mAb	Cell Signaling Technology, Danvers, MA, USA (2920)	1:1000	Anti-mouse IgG, HRP-linked Antibody	Cell Signaling Technology, Danvers, MA, USA (7076)	1:2000
PDK1 Antibody	Cell Signaling Technology, Danvers, MA, USA (3062)	1:1000	Anti-rabbit IgG, HRP-linked Antibody	Cell Signaling Technology, Danvers, MA, USA (7074)	1:2000
Phospho-PDK1 (Ser241) (C49H2) Rabbit mAb	Cell Signaling Technology, Danvers, MA, USA (3438)	1:1000	Anti-rabbit IgG, HRP-linked Antibody	Cell Signaling Technology, Danvers, MA, USA (7074)	1:2000
PTEN (D4.3) XP^®^ Rabbit mAb	Cell Signaling Technology, Danvers, MA, USA (9188)	1:1000	Anti-rabbit IgG, HRP-linked Antibody	Cell Signaling Technology, Danvers, MA, USA (7074)	1:2000
p70 S6 Kinase (49D7) Rabbit mAb	Cell Signaling Technology, Danvers, MA, USA (2708)	1:1000	Anti-rabbit IgG, HRP-linked Antibody	Cell Signaling Technology, Danvers, MA, USA (7074)	1:2000
Phospho-p70 S6 Kinase (Thr389) (1A5) Mouse mAb	Cell Signaling Technology, Danvers, MA, USA (9206)	1:500	Anti-mouse IgG, HRP-linked Antibody	Cell Signaling Technology, Danvers, MA, USA (7076)	1:2000
Phospho-Src Family (Tyr416) (D49G4) Rabbit mAb	Cell Signaling Technology, Danvers, MA, USA (6943)	1:1000	Anti-rabbit IgG, HRP-linked Antibody	Cell Signaling Technology, Danvers, MA, USA (7074)	1:2000
Src (36D10) Rabbit mAb	Cell Signaling Technology, Danvers, MA, USA (2109)	1:1000	Anti-rabbit IgG, HRP-linked Antibody	Cell Signaling Technology, Danvers, MA, USA (7074)	1:2000
Cleaved Caspase-9 (Asp315) Antibody (Human Specific)	Cell Signaling Technology, Danvers, MA, USA (9505)	1:5000	Anti-rabbit IgG, HRP-linked Antibody	Cell Signaling Technology, Danvers, MA, USA (7074)	1:2000
Caspase-9 (C9) Mouse mAb	Cell Signaling Technology, Danvers, MA, USA (9508)	1:1000	Anti-mouse IgG, HRP-linked Antibody	Cell Signaling Technology, Danvers, MA, USA (7076)	1:2000
Cleaved Caspase-3 (Asp175) Rabbit mAb	Cell Signaling Technology, Danvers, MA, USA (9661)	1:300	Anti-rabbit IgG, HRP-linked Antibody	Cell Signaling Technology, Danvers, MA, USA (7074)	1:2000
Caspase-3 (D3R6Y) Rabbit mAb	Cell Signaling Technology, Danvers, MA, USA (14220)	1:1000	Anti-rabbit IgG, HRP-linked Antibody	Cell Signaling Technology, Danvers, MA, USA (7074)	1:2000
Phospho-FAK (Tyr397) (D20B1) Rabbit mAb	Cell Signaling Technology, Danvers, MA, USA (8556)	1:1000	Anti-rabbit IgG, HRP-linked Antibody	Cell Signaling Technology, Danvers, MA, USA (7074)	1:2000
Cleaved Caspase-7 (Asp198) (D6H1) Rabbit mAb	Cell Signaling Technology, Danvers, MA, USA (8438)	1:400	Anti-rabbit IgG, HRP-linked Antibody	Cell Signaling Technology, Danvers, MA, USA (7074)	1:2000
Caspase-7 (D2Q3L) Rabbit mAb	Cell Signaling Technology, Danvers, MA, USA (12827)	1:1000	Anti-rabbit IgG, HRP-linked Antibody	Cell Signaling Technology, Danvers, MA, USA (7074)	1:2000
Cleaved PARP (Asp214) (D64E10) XP^®^ Rabbit mAb	Cell Signaling Technology, Danvers, MA, USA (5625)	1:1000	Anti-rabbit IgG, HRP-linked Antibody	Cell Signaling Technology, Danvers, MA, USA (7074)	1:2000
PARP (46D11) Rabbit mAb	Cell Signaling Technology, Danvers, MA, USA (9532)	1:1000	Anti-rabbit IgG, HRP-linked Antibody	Cell Signaling Technology, Danvers, MA, USA (7074)	1:2000
Anti-beta Actin Mouse Antibody	Abcam (ab6276)	1:10000	Anti-mouse IgG, HRP-linked	CST, Danvers, USA (7076)	1:2000

## Data Availability

Data used to generate PRKAR1B-AS2 lncRNA and PI3K-110α protein interaction score were derived from a publicly accessible repository that does not issue DOIs. These data can be generated using RPISeq (http://pridb.gdcb.iastate.edu/RPISeq/; accessed on 23 July 2020) and catRAPID Fragments (http://s.tartaglialab.com/page/catrapid_group; accessed on 23 July 2020) software.

## Supplementary Materials

The following are available online at https://www.mdpi.com/1422-0067/22/4/1882/s1, Figure S1: Analysis of Protein Kinase CAMP-Dependent Type I Regulatory Subunit Beta (PRKAR1B-AS2) long noncoding RNA, Figure S2: Cytotoxic effect of cisplatin and alpelisib on A2780-CP20 and SKOV3-TR cells, Figure S3: In vivo experiment.

## Abbreviations

AKT	Protein kinase B
DOPC	1,2-dioleoyl-sn-glycero-3-phosphocholine
lncRNA	Long non-coding RNA
mTOR	Mammalian target of rapamycin
OC	Ovarian cancer
OS	Overall survival
PDK1	Phosphoinositide dependent kinase 1
PI3K	Phosphoinositide 3-kinase
PIP2	Phosphatidylinositol-3,4-bisphosphate
PIP3	Phosphatidylinositol-3,4,5- triphosphate
PRKAR1B	Protein Kinase CAMP-Dependent Type I Regulatory Subunit Beta
PTEN	Phosphatase and tensin homolog
RIP	RNA immunoprecipitation
RPPA	Reverse phase protein array
siRNA	Small interfering RNA
TCGA	The cancer genome atlas
TSC1/2	Tuberous sclerosis complex 1 and 2
TUNEL	Terminal deoxynucleotidyl transferase dUTP nick-end labeling
