# Supplementary material for: PRKAR1B-AS2 Long Noncoding RNA Promotes Tumorigenesis, Survival, and Chemoresistance via the PI3K/AKT/mTOR Pathway
Source: Int J Mol Sci. 2021 Feb 13;22(4):1882. doi: 10.3390/ijms22041882 (PMC7918312; doi:10.3390/ijms22041882)
Supplement: Supplementary file 1 [file ijms-22-01882-s001.pdf]

A

```
ORIGIN
      1 tgcagccagg cactgtccgt cccgggacct acgcagctgc catggaagct ccgggtttcg
     61 caggagactg gaataagatc ggaataattc caacagatgc tccaaccatc cctcatggac
    121 cacacgcact cttggatgct gaagagcagg ctggggtctg gggcagcctt taggggctct
    181 tgggacccca aaggtgtcca gcttggatgt ctccagcaga tctttctgga aatctgggac
    241 accaaggagg agcacaaatg ctggtcttac tactaagcaa tatgtccctc ggatccaaag
    301 gagagcaggt gacctgggtg cagccacagc cagctgccac ctccctggag tccctcatga
    361 agcaatgcat tcagggcaga atccattgct gcctcagccc ccttcaaaga ccaggcccaa
    421 caccgcacca gctccttgga cactgcccat cttcccaggg agagtctcgt gctcagagag
    481 gctataaata tacgccggac agtcaagagt ttccgtcgcc aacagaagag ctgagcaaaa
    541 ggtgaaataa acatcccgtg gctgcaaatt aaaaaaaaaa aaaa
```

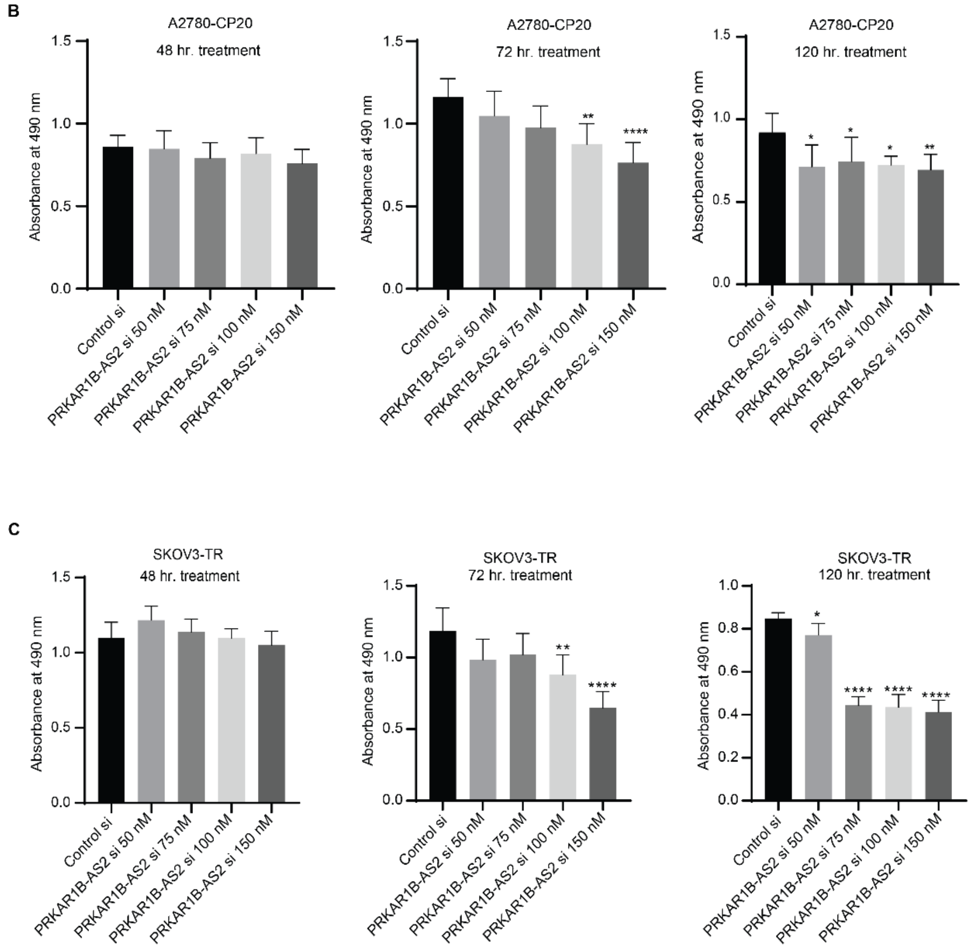

Supplementary Figure S1. Analysis of PRKAR1B-AS2 long noncoding RNA. (A) Nucleotide sequence of PRKAR1B-AS2. (B) Effect of PRKAR1B-AS2 knockdown on A2780-CP20 viability after 48, 72, and 120 h of treatment. (C) Effect of PRKAR1B-AS2 knockdown on SKOV3-TR viability after 48, 72, and 120 h of treatment.

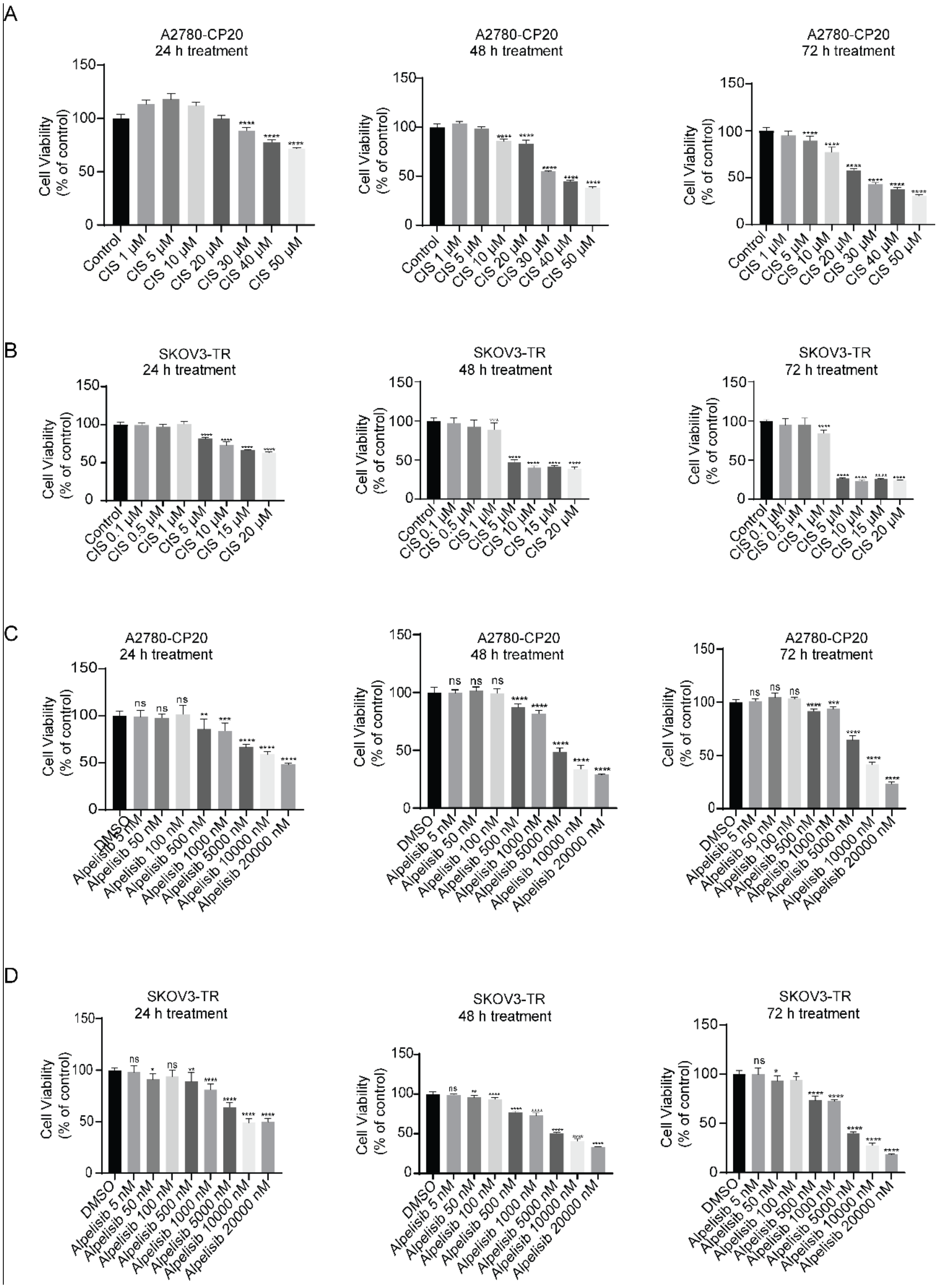

Supplementary Figure S2. Cytotoxic effect of cisplatin (CIS) and alpelisib on A2780-CP20 and SKOV3-TR cells. (A) Effect of cisplatin on A2780-CP20 viability. (B) Effect of cisplatin on SKOV3-TR viability. (C) Effect of alpelisib on A2780-CP20 viability. (D) Effect of alpelisib on SKOV3-TR viability.

A

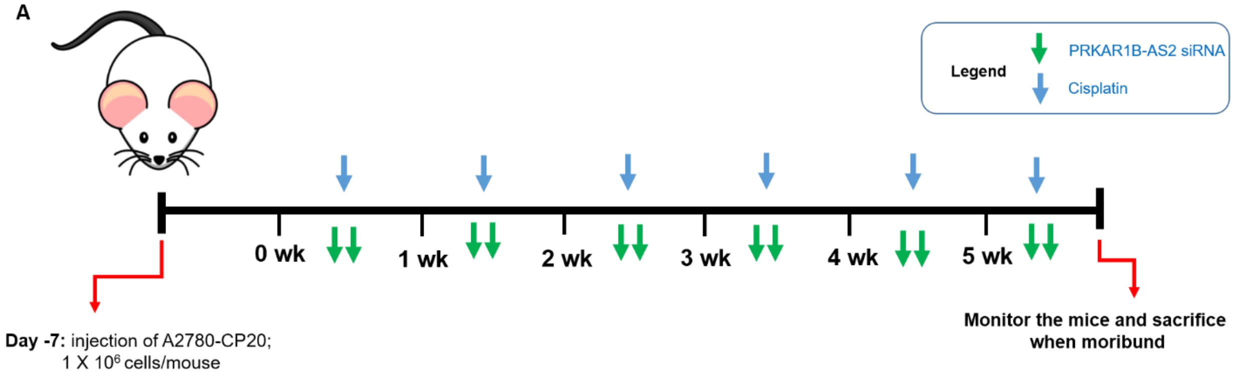

B

Group	No. of mice	Treatment	Dose
1	10	DOPC negative control siRNA (si-NC)	**Si-NC:** 200 µg/Kg, 2 x weekly, IV
2	10	DOPC PRKAR1B-AS2 siRNA	**Si-PRKAR1B-AS2:** 200 µg/Kg, 2 x weekly, IV
3	10	DOPC negative control siRNA (si-NC) + cisplatin	**Cisplatin:** 4mg/kg, 1 x weekly, IP
4	10	DOPC PRKAR1B-AS2 siRNA + cisplatin	**Cisplatin:** 4mg/kg, 1 x weekly, IP; **Si-PRKAR1B-AS2** 200 µg/Kg, 2 x weekly, IV

Supplementary Figure S3. *In vivo* experiment. (A) Design of the A2780-CP20 xenograft mouse models. (B) Treatment scheme for the four groups.
